# Structural Design and Optimization of a Resonant Micro-Accelerometer Based on Electrostatic Stiffness by an Improved Differential Evolution Algorithm

**DOI:** 10.3390/mi13010038

**Published:** 2021-12-28

**Authors:** Libin Huang, Qike Li, Yan Qin, Xukai Ding, Meimei Zhang, Liye Zhao

**Affiliations:** 1 School of Instrument Science and Engineering, Southeast University, Nanjing 210096, China; 220203477@seu.edu.cn (Q.L.); qhh12zhh@163.com (Y.Q.); ding.xk@seu.edu.cn (X.D.); 220193328@seu.edu.cn (M.Z.); liyezhao@seu.edu.cn (L.Z.); 2 Key Laboratory of Micro-Inertial Instruments and Advanced Navigation Technology, Ministry of Education, Nanjing 210096, China

**Keywords:** resonant accelerometer, electrostatic stiffness, structural design, differential evolution algorithm

## Abstract

This study designed an in-plane resonant micro-accelerometer based on electrostatic stiffness. The accelerometer adopts a one-piece proof mass structure; two double-folded beam resonators are symmetrically distributed inside the proof mass, and only one displacement is introduced under the action of acceleration, which reduces the influence of processing errors on the performance of the accelerometer. The two resonators form a differential structure that can diminish the impact of common-mode errors. This accelerometer realizes the separation of the introduction of electrostatic stiffness and the detection of resonant frequency, which is conducive to the decoupling of accelerometer signals. An improved differential evolution algorithm was developed to optimize the scale factor of the accelerometer. Through the final elimination principle, excellent individuals are preserved, and the most suitable parameters are allocated to the surviving individuals to stimulate the offspring to find the globally optimal ability. The algorithm not only maintains the global optimality but also reduces the computational complexity of the algorithm and deterministically realizes the optimization of the accelerometer scale factor. The electrostatic stiffness resonant micro-accelerometer was fabricated by deep dry silicon-on-glass (DDSOG) technology. The unloaded resonant frequency of the accelerometer resonant beam was between 24 and 26 kHz, and the scale factor of the packaged accelerometer was between 54 and 59 Hz/g. The average error between the optimization result and the actual scale factor was 7.03%. The experimental results verified the rationality of the structural design.

## 1. Introduction

As a kind of micro-electro-mechanical system (MEMS) accelerometer, a silicon resonant micro-accelerometer has the advantages of a direct digital signal output, high sensitivity, high resolution, wide dynamic range, strong anti-interference ability, and good stability [1,2,3,4,5]. It has the advantage of being useable to develop a higher-precision microelectromechanical accelerometer. The electrostatic stiffness resonant micro-accelerometer combines electrostatic stiffness with the resonance principle. The resonant frequency is affected by the change in electrostatic stiffness, which greatly reduces the dependence of the device performance on processing errors [6].

At present, there are two main types of resonant accelerometers based on electrostatic stiffness: in-plane and out-of-plane [7,8,9,10] detection. The in-plane electrostatic stiffness resonant micro-accelerometer mainly has two structural forms: one is a structure in which the proof mass and the resonator are independent [6,11,12,13,14,15,16,17], and the other is a structure in which the proof mass is a component of the resonator [18,19,20]. The first type of accelerometer is composed only of a double-ended tuning fork (DETF) and two proof masses. In terms of structure, the two proof masses and the resonator are independent of each other. The two beams of the DETF are electrically connected to the two masses by means of small parallel plate capacitors. This structure is simple and implementable. In the second type of accelerometer, the proof mass is an integral part of the resonator. The reverse arrangement of the two resonators is realized by the reverse arrangement of the electrostatic negative stiffness plate capacitor. The acceleration is calculated by the difference in the frequency of the two resonators. Each resonator is composed of two proof masses, and the in-phase and anti-phase vibration modes of the two proof masses are used for sensitive mode and modulation mode, respectively. Although the difficulty of designing sensitive structures is increased, the multiplexing of proof masses and resonators can better save the layout of sensitive structures.

The structure’s performance is determined by the structure itself, so the design and improvement of the micro-accelerometer structure have become one of the hot directions in the field of micro-accelerometer research. There are two main ideas for the structural design of the micro-accelerometer. One is to verify the rationality of the structural design by simulating the structure with finite element analysis software based on rich design experience and experiments. The relationship between performance indexes and key structural parameters is investigated through simulation. The optimization of the structural parameter values is realized by comprehensive consideration. This method can design structures that meet certain performance requirements, but it is difficult to find structures that meet the optimal performance. The other is to establish the mathematical model of the optimization problem according to the structural optimization theory and seek the optimal structural parameters that meet some design requirements under certain constraints. For example, Pei et al. [21] combined the zero-order method and the gradient search method to complete the coarse and exact optimization. The scalar factor of the optimized quartz beam accelerometer improved 16%, which improved the performance of the accelerometer. Wang and Zhao et al. [22] aimed at the piezoelectric accelerometer with high sensitivity. The beam configuration of the accelerometer with high sensitivity and low stress characteristics is obtained by means of a genetic algorithm. Wang et al. [23] used genetic algorithms to optimize the design of the flexible structure of the mechanically amplified MEMS accelerometer, which greatly improved the bandwidth and sensitivity of the accelerometer. In Pak’s research [24], a sensor noise model was developed for two MEMS accelerometers with the same topology, and the noise performance of the accelerometer was improved using the MOEA/D evolution algorithm optimization. Zhang and Shi [25] obtained the final optimized model by the NSGA-II algorithm using a combination of OSF-based and Kriging agent models. The optimized accelerometer size was reduced by 29.33%, and its resonant frequency and sensitivity were improved.

Based on the first type of accelerometer, we designed an in-plane electrostatic stiffness resonant micro-accelerometer. The accelerometer adopts a one-piece proof mass structure; two double-folded beam resonators are symmetrically distributed inside the proof mass. Only one displacement of the proof mass is introduced under acceleration, which reduces the influence of the processing error on the accelerometer performance. The two resonators form a differential structure, which can reduce the impact of common-mode errors. The structure realizes the separation of electrostatic stiffness introduction and resonant frequency detection, which is conducive to the decoupling of accelerometer signals and simplifies the design of accelerometer circuits.

The differential evolution algorithm is introduced to optimize the scale factor of the accelerometer. An improved differential evolution algorithm was developed to save the better individuals through the principle of last elimination and assign the most suitable algorithm parameters to the surviving individuals in order to stimulate the ability of the offspring individuals to find the global optimum. While maintaining the global optimality, the complexity of the algorithm is reduced. The parameters achieved the optimal configuration, and the structural optimization of the accelerometer was deterministically realized.

## 2. Structural Design of a Resonant Micro-Accelerometer Based on Electrostatic Stiffness

### 2.1. Overall Structure Design

**Figure 1:**
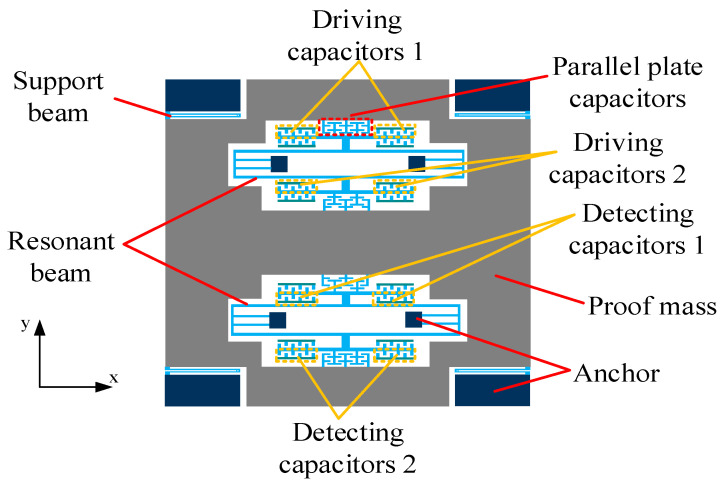
Schematic diagram of the overall structure of the accelerometer.

The overall structure of the new resonant micro-accelerometer based on electrostatic stiffness is shown in Figure 1. Two perfectly symmetrical double-folded beam resonators are contained in the accelerometer. The driving and detection of a single resonant beam are performed by two sets of comb capacitors, respectively, and the parallel plate capacitor is used only to adjust the resonant beam stiffness. The above capacitor design realizes the separation of electrostatic stiffness introduction and resonant frequency detection. During operation, the resonant beam is connected to the carrier signal, and the fixed driving comb capacitor is connected to the reverse AC voltage with DC bias to drive the resonant beam to the resonant state. Another DC voltage is applied to the proof mass to generate the voltage difference of the parallel plate capacitor, which introduces electrostatic negative stiffness for the tuning fork beam. Each resonator provides two sets of driving combs and detecting combs. One set of resonator driving combs is connected to voltage $V_{a1}=V_{c}sin\omega t+V_{d}$, and the other set is connected to $V_{a2}=-V_{c}sin\omega t+V_{d}$. Thus, $V_{a1}^{2}-V_{a2}^{2}\propto sin\omega t$, and the driving force is a sinusoidal harmonic force. The accelerometer adopts a one-piece proof mass structure. Two resonators are symmetrically distributed inside the proof mass to form a differential structure, reducing the influence of common-mode errors. The proof mass only introduces one displacement under the action of acceleration, which reduces the influence of manufacturing errors on the performance of the accelerometer.

Since the accelerometer proof mass adopts a one-piece structure, when there is no acceleration, the proof mass is subjected to two electrostatic forces of equal magnitude and opposite direction, the proof mass is in a static initial position, the electrostatic stiffnesses introduced by two resonators are equal. The resonant frequencies of two resonators are equal and the output of the accelerometer is zero. When the accelerometer is sensitive to acceleration, the proof mass is displaced under the action of inertial force. The gap of the parallel plate capacitors between the mass and a resonator increase, and the electrostatic stiffness decreases, which increases the resonant frequency. At the same time, the gap of the parallel plate capacitors between the proof mass and the other resonator reduces, and the electrostatic stiffness increases, which reduces the resonant frequency. The resonant frequency difference of two resonators is used as the output of the accelerometer, which is approximately linear with the input acceleration.

### 2.2. Theoretical Analysis

For dynamic analysis of a resonator, the vibration equation of a single resonant beam can be expressed as [13,14]

(1) $$m\ddot{y}+c\dot{y}+ky=F_{d}+F_{e}$$

where *m* is the effective mass of the resonant beam vibrating transversely, *c* is the vibration damping coefficient, *k* is the effective mechanical stiffness of the resonant beam, y is the resonant beam vibration mode coordinate, $F_{d}$ is the driving force generated by the driving comb capacitor in the resonant beam vibration direction, and $F_{e}$ is the electrostatic force generated by the parallel plate capacitor in the resonant beam vibration direction.

Substituting specific expressions of $F_{d}$ and $F_{e}$ into Equation (1), ignoring higher terms and combining like terms, we have [13,26]

(2) $$m\ddot{y}+c\dot{y}+\left(k-k_{e}\right)y=\frac{N\varepsilon h}{2d_{0}}V_{a}^{2}-\frac{\varepsilon AV_{s}^{2}}{2g_{0}^{2}}$$

where *N* is the number of driving comb capacitor pairs, ε is the dielectric constant, *h* is the effective thickness of the driving comb capacitor, $d_{0}$ is the gap of the driving comb capacitor pole plates, $V_{a}$ is the driving voltage of the comb structure, *A* = $N_{S}hl$ is the orthogonal area of the parallel plate structure, $N_{S}$ is the number of parallel plate capacitor pairs, l is the effective length of each pair of parallel plates, $g_{0}$ is the static initial gap of a single parallel plate capacitor, $\;V_{s}$ is the potential difference between the resonant beam and the parallel plate, and $k_{e}=\frac{\varepsilon AV_{s}^{2}}{g_{0}{}^{3}}$ is the electrostatic stiffness. When a potential difference exists between the resonant beam and the proof mass, the electrostatic negative stiffness is generated to make the resonant beam stiffness weaker and reduce its resonant frequency.

Let $y_{1}$ be the displacement corresponding to the change in the y-direction of the resonant beam under the action of electrostatic force when the acceleration is zero. $\Delta y_{1}$ is the displacement of the resonant beam in the *y*-direction relative to $y_{1}$ under the action of electrostatic force when the acceleration is not zero. Unlike other accelerometers, this accelerometer adopts a single proof mass structure. When there is no acceleration, the proof mass is subjected to two electrostatic forces of equal magnitude and opposite directions. In this case, the displacement of the proof mass-support beam system in the *y*-direction is 0. Let $\Delta y_{2}$ be the displacement of the proof mass-support beam system in the y-direction when the acceleration is not 0. When the accelerometer is operating, a DC voltage *Vs* is applied at the proof mass anchor, the driving comb is connected to $V_{a1}=V_{c}sin\omega t+V_{d}$, and the resonant beam is connected to a square wave signal *V_f_*. The output frequency $f_{e1}$ can be expressed as [14]

(3) $$f_{e1}=\frac{1}{2\pi }\sqrt{\frac{k-\frac{\varepsilon AV_{s}^{2}}{{\left(g_{0}+\Delta y_{2}-y_{1}+\Delta y_{1}\right)}^{3}}}{m}}$$

Similarly, the output frequency $f_{e2}$ of the other resonator can be expressed as

(4) $$f_{e2}=\frac{1}{2\pi }\sqrt{\frac{k-\frac{\varepsilon AV_{s}^{2}}{{\left(g_{0}-\Delta y_{2}-y_{1}-\Delta y_{1}\right)}^{3}}}{m}}$$

Through calculation and simplification [13,26], we can obtain

(5) $$\left\{ \begin{array}{ll} f_{e1} & =\frac{1}{2\pi }\sqrt{\frac{k-\frac{\varepsilon AV_{S}^{2}}{g_{0}^{3}{\left(1+\frac{\Delta y_{2}}{g_{0}}\right)}^{3}}}{m}}=f_{0}\sqrt{1-\frac{\beta }{{\left(1+\alpha \right)}^{3}}}\\ & \approx f_{0}(\sqrt{1-\beta }+\frac{3}{2}\frac{\beta }{\sqrt{1-\beta }}\alpha -\frac{3}{4}(\frac{3\beta^{2}}{\sqrt{{\left(1-\beta \right)}^{3}}}+\frac{8\beta }{\sqrt{\left(1-\beta \right)}})\alpha^{2}+o(\alpha^{2}))\\ f_{e2} & =\frac{1}{2\pi }\sqrt{\frac{k-\frac{\varepsilon AV_{S}^{2}}{g_{0}^{3}{\left(1-\frac{\Delta y_{2}}{g_{0}}\right)}^{3}}}{m}}=f_{0}\sqrt{1-\frac{\beta }{{\left(1-\alpha \right)}^{3}}}\\ & \approx f_{0}(\sqrt{1-\beta }-\frac{3}{2}\frac{\beta }{\sqrt{1-\beta }}\alpha -\frac{3}{4}(\frac{3\beta^{2}}{\sqrt{{\left(1-\beta \right)}^{3}}}+\frac{8\beta }{\sqrt{\left(1-\beta \right)}})\alpha^{2}+o(\alpha^{2})) \end{array}\right.$$

The frequency difference of the two resonators is

(6) $$\Delta f=f_{e1}-f_{e2}\approx f_{0}\frac{3\beta }{\sqrt{1-\beta }}\alpha$$

where $f_{0}$ is the unloaded resonant frequency of the beam, *β* is the stiffness ratio, and their expressions are as follows [27]:

(7) $$f_{0}=\frac{1}{2\pi }\sqrt{\frac{k}{m}}=\frac{1}{2\pi }\sqrt{\frac{16.539Ew^{3}}{L^{3}\left(0.397\rho A_{l}+\rho A_{f}\right)}},\;\beta =\frac{k_{e}}{k},\;\Delta y_{2}=\frac{m_{s}\cdot a}{k_{s}-2k_{e}},\;\alpha =\frac{\Delta y_{2}}{g_{0}}=\frac{m_{s}a}{g_{0}\left(k_{s}-2k_{e}\right)}$$

where *E* is the modulus of elasticity of silicon; *ρ* is the density of silicon; *L*, *w*, and *h* are the length, width, and thickness of the resonant beam, respectively; $A_{l}$ *= wL* is the surface area of the beam; $A_{f}$ is the surface area of the additional proof mass; and $m_{s}$ is the mass of the proof mass.

The scale factor (SF) is an important indicator for assessing the performance of an accelerometer, and is expressed as

(8) $$\begin{array}{ll} SF\approx \frac{\delta \Delta f}{\delta a}g_{n}\approx 3f_{0}\frac{\beta }{\sqrt{1-\beta }}\cdot \frac{m_{s}}{g_{0}\left(k_{s}-2k_{e}\right)}g_{n} & =\frac{3k_{e}f_{0}}{\sqrt{k\left(k-k_{e}\right)}}\cdot \frac{m_{s}}{g_{0}\left(k_{s}-2k_{e}\right)}g_{n}\\ & =\frac{1}{2\pi }\frac{3k_{e}}{\sqrt{m\left(k-k_{e}\right)}}\cdot \frac{m_{s}}{g_{0}\left(k_{s}-2k_{e}\right)}g_{n} \end{array}$$

where $g_{n}$ is the value of gravitational acceleration.

## 3. Structural Optimization Design by the Improved Differential Evolution Algorithm

### 3.1. Optimization Objectives

The structure size parameter is brought into Equation (8) to obtain

(9) $$SF=\frac{1}{2\pi }\frac{3\varepsilon AV_{s}^{2}}{g_{0}^{3}\sqrt{\left(0.397\cdot \rho wLh+\rho A_{f}h\right)\times \left(16.539\cdot \frac{Ew^{3}h}{L^{3}}-\frac{\varepsilon AV_{s}^{2}}{g_{0}^{3}}\right)}}\cdot \frac{\left(\rho w_{2}L_{2}h-\rho A_{t}h+\rho A_{p}h\right)}{g_{0}\left(\frac{Ew_{1}^{3}h}{2L_{1}^{3}}-2\frac{\varepsilon AV_{s}^{2}}{g_{0}^{3}}\right)}\cdot g_{n}$$

where $w_{2}$ and $L_{2}$ are the maximum values in the length and width directions of the proof mass, $A_{t}$ is the area of the hollowed-out part of the proof mass, and $A_{p}$ is the area of the parallel plate structure inside the proof mass, $L_{1}$ is the length of the support beam, and $w_{1}$ is the width of the support beam. There are many parameters that affect the accelerometer scale factor in Equation (9), but the key parameters are DC voltage $V_{S}$; resonant beam length L and width w; support beam length $L_{1}$ and width $w_{1}$; and the initial gap between parallel plates $g_{0}$.

It can be seen from Equation (9) that $V_{s}$ is positively correlated with *SF*; however, due to the pull-in effect of the parallel plate capacitor structure in the accelerometer, the DC voltage $V_{s}$ connected to the proof mass should be less than the pull-in voltage $V_{s}^{\prime }$. It is also known from the literature [13,17,26,28] that the pull-in voltage $V_{s}^{\prime }$ is related to the size parameter, and that $V_{s}^{\prime }$ is negatively related to the range. Considering the range and structural stability, the pull-in voltage $V_{s}^{\prime }$ is controlled between 20 and 30 V [17]. 

Therefore, the range of $V_{s}$ is determined as $5\leq V_{s}\leq 15$ V $\left(V_{s}<0.8\cdot V_{s}^{\prime }\right)$. Considering the size of the package housing, $w_{2}$ is taken as 2800 μm and $L_{2}$ as 3800 μm.

Considering the existing manufacturing technology, pull-in voltage, scale factor, nonlinearity, and other factors, the optimization problem is shown in Equation (10):

(10) $$\min f\left(x\right)=-SF\;s.t.\;3<g_{0}<4.5,\;1100<L<1300,\;7.5<w<10,\;500<L_{1}<600,\;9<w_{1}<12,\;24,000<f_{0}<30,000,\;20<k_{s}<36,\;0<k_{e}<4,\;5\leq V_{s}\leq 15\;\mathrm{and}\;V_{s}<0.8\cdot V_{s}^{\prime }$$

where the units of $k_{e}$ and $k_{s}$ in the above equation are measured in N/m, and the other length units are measured in μm. The goal of structural optimization design is to obtain the optimal value of the scale factor under the above constraints. In the actual process of optimizing the accelerometer problem, the parameter dimension is 6 (length and width of the resonant beam; length and width of the support beam; parallel plate capacitor gap; and DC voltage $V_{S}$ (taking into account the pull-in effect)), corresponding to the above-given limitation factors. The other structural parameters of the accelerometer are shown in Table 1.

### 3.2. Standard DE

The DE (differential evolution) algorithm [29,30] was originally designed to solve the Chebyshev polynomial. The main idea is to use the differences between individuals in the population to make the next generation of individuals search the solution space to find the optimal solution. The main process includes initial population, mutation operation, crossover operation, and selection operation.

The steps for standard DE are as follows [30].

#### 3.2.1. Initialization

After determining the constraints on the number of populations *NP*, the maximum number of generations $G_{max}$ and the dimensionality of the problem *D*, the initial generation of individuals (vectors) within the population is initialized:

(11) $$x^{G}=(x_{\left(1\right)}^{G},x_{\left(2\right)}^{G},\ldots \ldots ,x_{\left(NP\right)}^{G})\;G=1,2,3,\ldots ,G_{\max}$$

where $x_{\left(i\right)}^{G}=\left[x_{\left(i,1\right)}^{G},x_{\left(i,2\right)}^{G},\ldots \ldots ,x_{\left(i,d\right)}^{G}\right]\;i=1,2,\ldots ,NP$.

The initial vector is randomly selected and can be represented by the following equation:

(12) $$x_{\left(i,j\right)}^{0}=x_{\left(i,jmin\right)}+rand\left(0,1\right)\left[x_{\left(i,jmax\right)}-x_{\left(i,jmin\right)}\right]\;j=1,2,\ldots ,D$$

where $x_{\left(i,jmin\right)}$ and $x_{\left(i,jmax\right)}$ are the limit range of the *j*-th parameter.

#### 3.2.2. Mutation Operation

In this step, the standard DE algorithm is described as the mutation of all target individuals in the population:

(13) $$\mathrm{DE}/\mathrm{rand}/1:\;v_{i}^{G}=x_{r1}^{G}+F\cdot \left(x_{r2}^{G}-x_{r3}^{G}\right)\;\mathrm{where},\;F\in \left(0,1\right)$$

The algorithm adopts the DE/rand/1 mutation strategy. In this step, each mutated individual is composed of a parent part and a mutated part. The mutated part is obtained by the difference of two randomly selected individuals from the parent population, except for the aforementioned parent individual. The individual obtained by the mutation operation is the mutation vector [31]. In addition, Price, Storn, and other studies have proposed various strategies, including DE/best/1 and DE/rand-to-best/1 [29,32].

#### 3.2.3. Crossover Operation

The mutant individuals generated in the previous generation are cross operated with their parents to generate test vectors. At least one element of the test vector comes from a mutated individual, which provides power for the next generation of population evolution.

(14) $$u_{\left(i,j\right)}=\left\{ \begin{array}{ll} v_{\left(i,j\right)}, & if\;(\mathrm{rand}(0,1)\leq CR)\;\mathrm{or}\;j=j_{rand}\;\\ x_{\left(i,j\right)}, & otherwise \end{array}\right.$$

where $j_{rand}$ = rand(0,1), and *CR* is the crossover rate, which is a key parameter in the differential evolution algorithm that reflects the differences between parent and offspring individuals.

#### 3.2.4. Selection Operation

After the crossover operation is completed, the objective function values of the test individuals u and x are used for one-to-one selection. For the minimization problem, the selection operation can be expressed as

(15) $$x_{i}^{G+1}=\left\{ \begin{array}{ll} u_{i}^{G+1}, & f\left(u_{i}^{G+1}\right)\leq f\left(x_{i}^{G}\right)\\ x_{i}^{G}\;, & otherwise \end{array}\right.$$

It is important to select the better vector to survive to the next generation by comparing the parent vector with the test vector. The above steps are then repeated until the number of evolutionary generations reaches $G_{max}$.

### 3.3. Improved Differential Evolution Algorithm

The DE optimization algorithm has the problems [31] of search stagnation and premature convergence in the application. The main reasons for this are that [33] (1) strict constraints may create an extremely narrow region in which the optimal objective function value exists, and that (2) it is of great significance to have a high searchability to leave out the local optimum when searching for the best results. Therefore, it is a necessary condition to have a strong global search ability to maintain population diversity. The performance of the DE algorithm mainly depends on several control parameters, including scale factor *F* [34], the crossover rate *CR*, population size *NP* [35,36], and the mutation strategy. 

Various self-adaptive DE algorithms have been proposed by many researchers. For example, Liu and Lampinen proposed a fuzzy adaptive DE algorithm [37], which uses a fuzzy logic controller to adjust the parameters *F* and *CR*. Qin et al. proposed the SaDE algorithm [38], where *F* and *CR* are adaptively adjusted based on a previous high-quality solution experience. Fan and Zhang proposed a differential evolution algorithm, CSA-SADE [39], with crossover strategy adaptation; this method can obtain suitable control parameters, mutation strategies, and crossover strategies at different stages of evolution. By optimizing the mutation strategy, Deng developed a new, improved DE algorithm based on the wavelet basis function [40], which realized the acceleration of convergence and the search for the global optimum.

The idea of RDE [41] is that when the parent is a better individual, a new individual is generated near it, then a mutation vector is generated near the parent vector through a smaller *F*, and a test vector is generated near the mutation vector through a larger *CR*. If the parent vector is poor, the mutation vector is generated by a larger *F* far away from the parent vector, and the smaller *CR* generates the test vector far away from the mutation vector. This balances the convergence and divergence of the search well, improves the search efficiency of the algorithm, and reduces unnecessary searches and the computational complexity of the algorithm.

Based on RDE and considering the globality and optimality of DE algorithm optimization, an improved algorithm, SAPRDE (self-adaptive population rank-based differential evolution), was developed. SAPRDE sorts the previous generation population during the evolution process, then preserves the good individuals through the final elimination principle. In addition, after population sorting and global parameter sorting, it maps the most suitable parameters, mutation rate *F* and crossover rate *CR* to each surviving individual, which stimulates the ability of offspring individuals to find the global optimum. The above is SAPRDE’s core idea. In this way, global optimality is maintained while algorithm complexity is reduced. Moreover, the parameters are optimally configured, and the optimization of the accelerometer scale factor is achieved. 

The initial population size ($NP_{max}$) of the SAPRDE algorithm is 15*D*–20*D*, and the termination size ($NP_{min}$) is 2*D*. For the DE algorithm, different mutation strategies will have different effects on the performance of the algorithm. The SAPRDE algorithm adopts the DE/best/1 strategy. At the beginning of evolution, the rich population makes the algorithm’s global search ability strong. On this basis, the adaptively adjusted mutation and crossover rates make the optimal evolution direction unrestricted.

If the mutated vector exceeds the specified range in the optimization process, it will be initialized again. Then, the program will continue to run. The main steps of SAPRDE are as follows. Record the information provided by the ranking in each round. One is to provide the basis for the parameter allocation for the population; the other is to provide information on the linear decrease in the population number *NP*. Then, the parameters are taken linearly in the interval and wait for allocation according to Equations (16) and (17).

(16) $$F_{j}=F_{min}+\left(F_{max}-F_{min}\right)\frac{S_{j}}{NP-1}$$

(17) $$CR_{j}=CR_{max}-\left(CR_{max}-CR_{min}\right)\frac{S_{j}}{NP-1}$$

(18) $$NP_{i}=NP_{max}-round\left[\frac{2i}{G_{max}}\left(NP_{max}-NP_{min}\right)\right],\;when\;i\leq \frac{G_{max}}{2}$$

**Figure 2:**
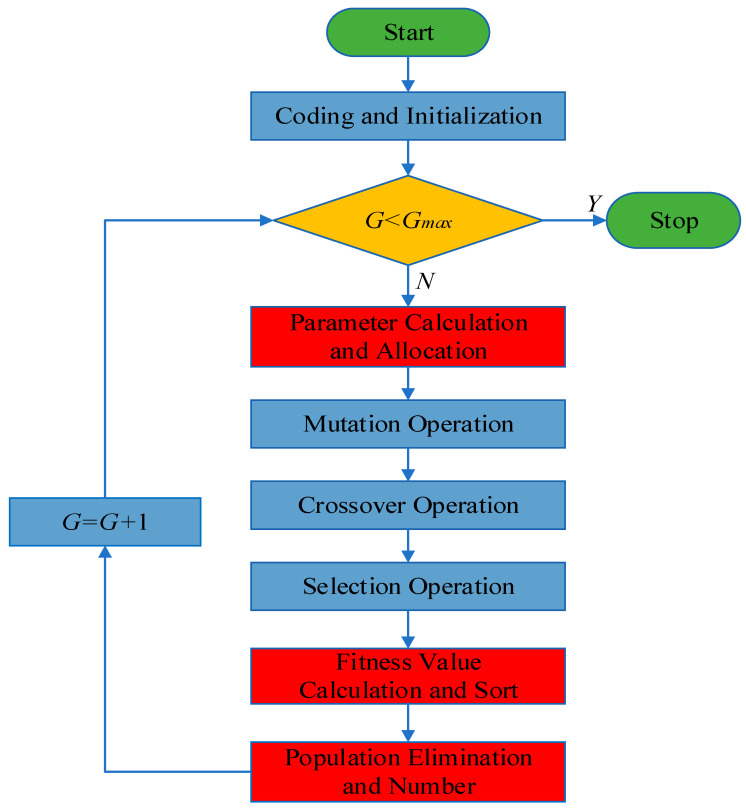
SAPRDE flow chart.

In Equations (16)–(18), *i* is the current evolutionary generation, $S_{j}$ is the ranking of the fitness of each individual in the population in each round, and $F_{j}$ and $CR_{j}$ are the parameters that are linearly allocated to the population during the execution process. Taking the interval of CR and F suggested in the article of Storn and Price as the standard, $F_{min}$, $F_{max}$, $CR_{min}$, and $CR_{max}$ are taken as the two ends of the interval (0.5,1), (0.8,1); the specific values optimize changes with the actual accelerometer parameters. On this basis, the algorithm uses the parameters to be assigned as calculated in Equations (16) and (17) to linearly assign the population. Figure 2 is the flow chart of the algorithm program. After the judgment operation between $sf\left(u_{i}^{G+1}\right)$ and $sf\left(x_{i}^{G}\right)$, the algorithm sorts the fitness of all surviving individuals in all populations at the time described above. After sorting, the ranking information is recorded. Obviously, the ranking information is used for the corresponding selection of the next round of parameter allocation. The algorithm eliminates bad individuals every few rounds, leaving the top-ranked individuals, so the ranking information also eliminates poor individuals. This is a method of RDE assignment. The number of populations in each round is shown in Equation (18). Then, the next round begins, and so on, until the termination conditions are met. It should be noted that the eliminated vector needs to be approximated before the elimination process is performed. All the improvement steps are marked in red in Figure 2. After the above steps, every control parameter in each round is assigned to the most suitable surviving individuals. This algorithm thusly realizes self-adaptive parameter assignment, eliminates bad individuals, and reduces unnecessary calculations.

### 3.4. Optimization Results

**Figure 3:**
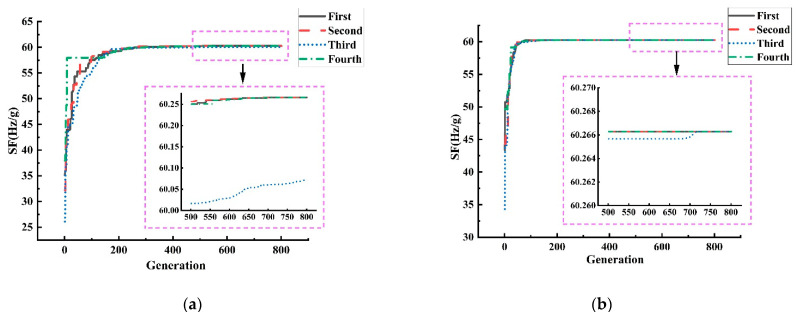
Comparison of optimization of two algorithms. (**a**) ordinary DE algorithm. (**b**) SAPRDE algorithm.

**Figure 4:**
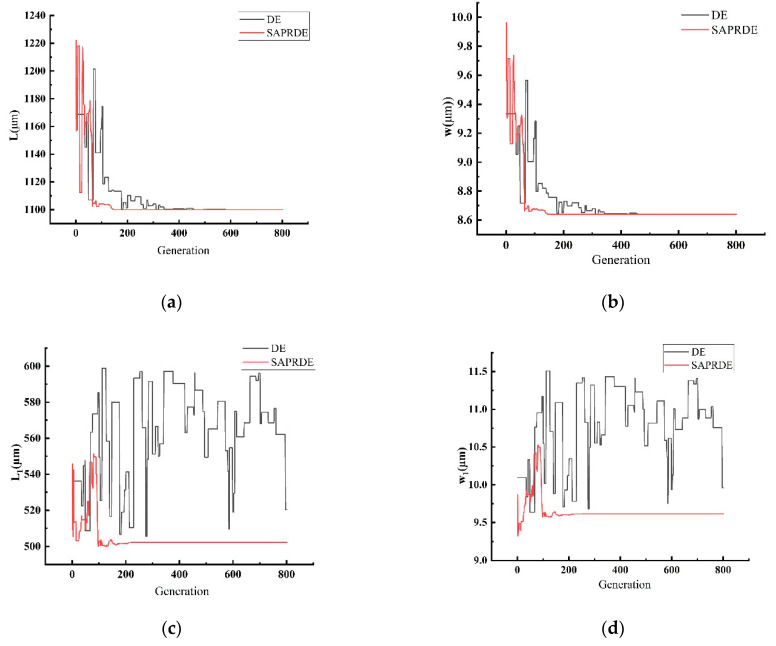

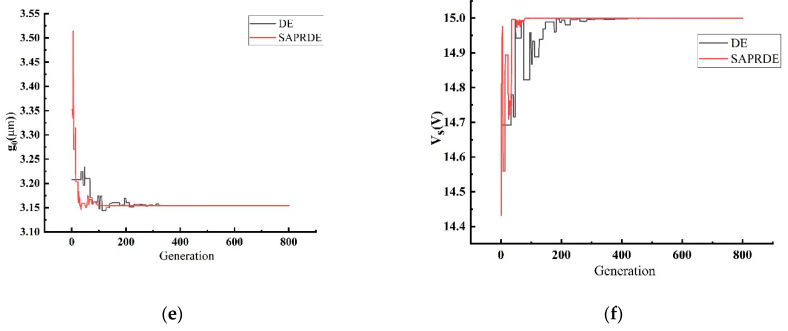
The optimization process of each parameter before and after algorithm improvement. (**a**) Length of the resonant beam L; (**b**) width of the resonant beam w; (**c**) length of support beam $L_{1}$; (**d**) width of support beam $w_{1}$; (**e**) gap of parallel plate capacitor $g_{0}$; (**f**) DC voltage $V_{s}$.

The optimization algorithm program was run in the software Matlab2020b. To reflect the effect of SAPRDE, we used the ordinary DE algorithm with the same strategy as SAPEDE for comparison. The algorithm parameters and condition settings of both were the same, except for the improved content proposed in this article, in the sense that both used the parameter interval suggested in Storn and Price [30]. Four results of the two algorithms can be randomly taken for comparison. In Figure 3, the general DE algorithm eventually reaches a stable state after 200–500 generations (generally 300 generations) of evolution. In the process of evolution, the algorithm reaches stability with too many evolutionary generations due to its slow speed of searching for good individuals. The value of the stable state did not ultimately converge to a fixed value: it converged to the local optimum and lacked the motivation to jump out of the local area. In contrast, the four runs in Figure 3b demonstrate the good repeatability of the SAPRDE algorithm, as the results of several runs were relatively consistent. The algorithm basically reached a stable state near the 100th generation and evolved to stabilize at 200–300 generations. After improvement, the number of iterations to find the optimal parameters was greatly reduced, and the success rate of finding the optimal solution was also higher. The results prove that the above-mentioned self-adaptive strategy increases the diversity of the population. Figure 4 shows the change curve of each specific parameter in the study of the evolution process.

As shown in Figure 4, it is obvious that the performance of SAPRDE is better than that of the ordinary DE algorithm in the optimization of the accelerometer parameters. The ordinary DE algorithm undergoes a long evolutionary process in parameter optimization. For example, the support beam length $L_{1}$ and width $w_{1}$ were still unstable at nearly 800 generations. On the contrary, the SAPRDE algorithm quickly searches for the optimal values of each parameter and quickly enters a stable state. The convergence values of $L,\;w,\;L_{1},\;w_{1}$, $g_{0}$, and $\;V_{S}$ were 1100 μm, 8.64 μm, 502.26 μm, 9.61 μm, 3.15 μm, and 15 V, respectively.

**Figure 5:**
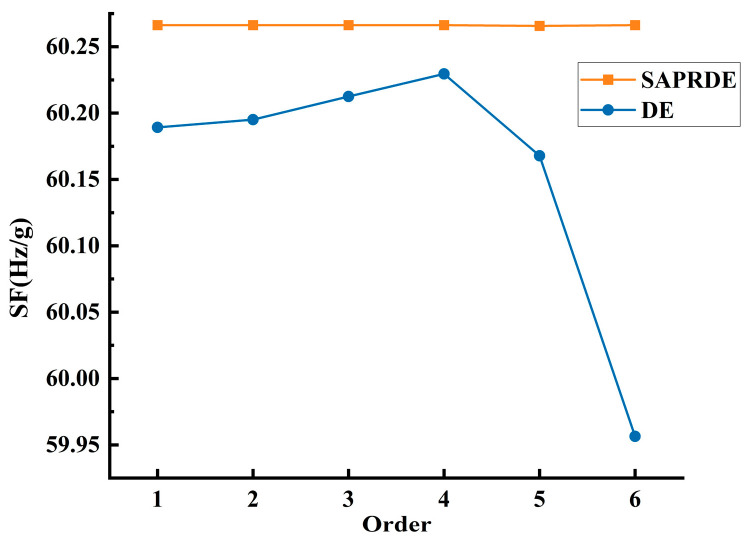
Convergence value at 400 generations of 6 random evolutions.

Figure 5 shows the evolution value of four hundred generations in six random runs. Unlike the ordinary DE algorithm, the result of SAPRDE was almost identical, which shows the good repeatability of the improved algorithm. In addition, we can see that the final value of the ordinary DE algorithm did not achieve the optimal effect every time. This is because the algorithm cannot be adjusted in time when it falls into local optimization, resulting in the evolution result not reaching the ideal optimization. In terms of time complexity, SAPRDE also had a running time reduction of 25% compared to the ordinary DE algorithm, which also verifies that the method of selecting excellent individual survival of the fittest reduces the overall computational complexity of the algorithm. The optimized result of the scale factor was 60.27 Hz/g; the unloaded resonant frequency of the resonator beam was 24.148 kHz.

## 4. Experiment

**Figure 6:**
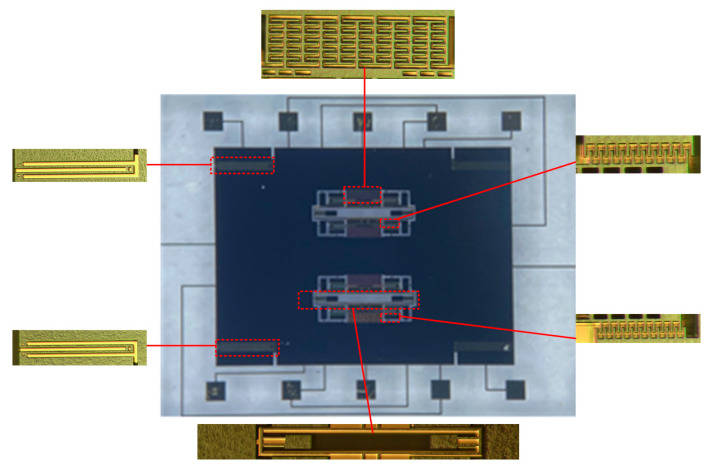
Photographs of the fabricated accelerometer.

The resonant micro-accelerometer based on electrostatic stiffness was fabricated by the deep dry silicon-on-glass (DDSOG) process. Photographs of the fabricated resonant accelerometer taken under the microscope are shown in Figure 6.

**Figure 7:**
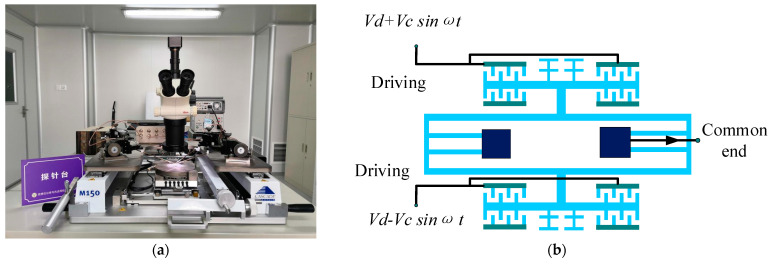
Open-loop test. (**a**) Probe station. (**b**) Accelerometer open-loop test pinout.

With the help of the probe station (Figure 7a), an open-loop test of the accelerometer was conducted. The outer fixed combs of the upper resonant beam and the inner fixed combs of the lower resonant beam are the driving end, and the opposite is the detection end. The two sides of the resonator are connected with a DC biased AC driving voltage to drive the resonator to the reverse working mode. The common terminal current is drawn at the anchor. The test leads are shown in Figure 7b.

All the accelerometers on the wafer were tested in the open-loop mode. The unloaded resonant frequencies of all the accelerometers were between 24 and 26 kHz. The test data of 10 accelerometers are shown in Table 2. Due to manufacturing errors, the resonant frequency of the resonator fluctuates, and there is a difference between the resonant frequencies of the two resonators of the same accelerometer. In general, the measured resonant frequencies of the fabricated accelerometer were basically consistent with the theoretical values.

**Figure 8:**
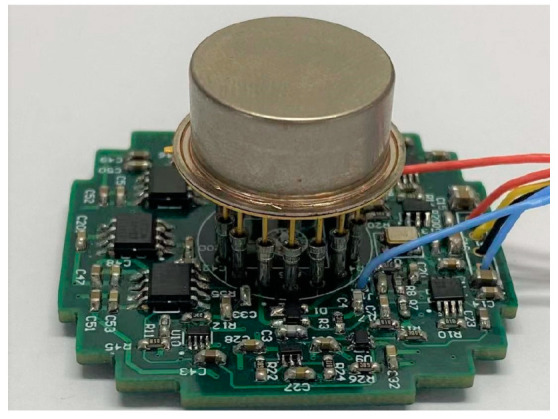
Packaged accelerometer and closed-loop circuits.

The scale factor test of five packaged accelerometers was performed through closed-loop circuits (Figure 8). With the 15 V detection voltage provided, the scale factor of accelerometers is shown in Table 3. It can be seen from the test data that the average error between actual scale factor and the result of the optimized design of five packaged accelerometers was 7.03%. The actual scale factor of the packaged accelerometers was basically consistent with the result of the optimized design, taking into account factors such as manufacturing and packaging errors.

## 5. Conclusions

This paper designed a resonant micro-accelerometer based on electrostatic stiffness. The working principle of the accelerometer was analyzed, and the expression of the scale factor was deduced. The mathematical model for the optimal design of a specific accelerometer structure was determined. An improved differential evolution algorithm, SAPRDE, was developed to optimize the accelerometer scale factor. The improved algorithm not only maintained the global optimality of the scale factor, but also reduced the complexity of the algorithm. The optimization results show that the SAPRDE algorithm has obvious advantages over the ordinary DE algorithm in terms of global search and computing time. The optimized accelerometer was fabricated by the DDSOG process. The unloaded resonant frequency of the fabricated accelerometer resonant beam was between 24 and 26 kHz, and the scale factor of the packaged accelerometers was between 54 and 59 Hz/g, which met the design and optimization expectations. The results showed that the SAPRDE algorithm optimization was in accordance with the structural characteristics of the resonant micro-accelerometer based on electrostatic stiffness.

## Figures and Tables

**Table 1:** Structural parameters of the accelerometer.

Parameter	Values	Units
Structural layer thickness	60	μm
Driving comb length	20	μm
Driving comb width	4	μm
Detecting comb length	20	μm
Detecting comb width	4	μm
Parallel plate capacitor length	25	μm
Parallel plate capacitor width	4	μm
Comb frame length	700	μm
Comb frame width	20	μm
Distance between two resonant beams	100	μm

**Table 2:** Unloaded resonant frequency of accelerometer resonator (kHz).

Accelerometer Number	1	2	3	4	5	6	7	8	9	10
Upper resonator	24.64	25.67	24.77	25.93	25.57	25.63	25.89	25.36	25.41	25.23
Lower resonator	24.69	25.78	24.86	25.98	25.53	25.64	25.87	25.43	25.48	25.29

**Table 3:** Scale factor and error of packaged accelerometers.

Accelerometer Number	1	2	3	4	5
Scale factor (Hz/g)	54.23	55.46	55.94	56.36	58.17
Error (%)	10.02	7.98	7.18	6.49	3.48
